# Epiisopilosine alkaloid has activity against *Schistosoma mansoni* in mice without acute toxicity

**DOI:** 10.1371/journal.pone.0196667

**Published:** 2018-05-11

**Authors:** Maria A. Guimarães, Rosimeire N. de Oliveira, Rebeca L. de Almeida, Ana C. Mafud, Ana L. V. Sarkis, Rayane Ganassin, Marcos P. da Silva, Daniel B. Roquini, Leiz M. Veras, Tânia C. H. Sawada, Cristina D. Ropke, Luis A. Muehlmann, Graziella A. Joanitti, Selma A. S. Kuckelhaus, Silmara M. Allegretti, Yvonne P. Mascarenhas, Josué de Moraes, José R. S. A. Leite

**Affiliations:** 1 Núcleo de Pesquisa em Biodiversidade e Biotecnologia, BIOTEC, Universidade Federal do Piauí, Parnaíba, Piauí, Brazil; 2 Phytobios Pesquisa Desenvolvimento e Inovação LTDA, Parnaíba, Piauí, Brasil; 3 Programa de Pós-graduação em Biotecnologia, RENORBIO, Ponto Focal Universidade Federal do Piauí, Teresina, Piauí, Brazil; 4 Departamento de Biologia Animal, Instituto de Biologia, Universidade Estadual de Campinas, Campinas, São Paulo, Brazil; 5 Instituto de Física de São Carlos, Universidade de São Paulo, São Carlos, São Paulo, Brazil; 6 Dept Medical Parasitology and Infection Biology, Swiss Tropical and Public Health Institute, Basel, Switzerland; 7 Faculdade de Medicina, Universidade de Brasília, UNB, Campus Dacy Ribeiro, Brasília, Distrito Federal, Brazil; 8 Laboratório de Nanobiotecnologia, Instituto de Biologia, Universidade de Brasília, UNB, Campus Dacy Ribeiro, Brasilia, Distrito Federal, Brazil; 9 Núcleo de Pesquisa em Doenças Negligenciadas, Universidade Guarulhos, Guarulhos, São Paulo, Brazil; 10 Faculdade de Ceilandia, Universidade de Brasília, UNB, Brasília, Distrito Federal, Brazil; Alexandria University, EGYPT

## Abstract

Schistosomiasis is a disease caused by parasites of the genus *Schistosoma*, currently affecting more than 200 million people. Among the various species of this parasite that infect humans, *S*. *mansoni* is the most common. Pharmacological treatment is limited to the use of a single drug, praziquantel (PZQ), despite reports of parasite resistance and low efficacy. It is therefore necessary to investigate new potential schistosomicidal compounds. In this study, we tested the efficacy of epiisopilosine (EPIIS) in a murine model of schistosomiasis. A single dose of EPIIS (100 or 400 mg/kg) administered orally to mice infected with adult *S*. *mansoni* resulted in reduced worm burden and egg production. The treatment with the lower dose of EPIIS (100 mg/kg) significantly reduced total worm burden by 60.61% (*P* < 0.001), as well as decreasing hepatosplenomegaly and egg excretion. Scanning electron microscopy revealed morphological changes in the worm tegument after treatment. Despite good activity of EPIIS in adult *S*. *mansoni*, oral treatment with single dose of EPIIS 100 mg/kg had only moderate effects in mice infected with juvenile *S*. *mansoni*. In addition, we performed cytotoxicity and toxicological studies with EPIIS and found no *in vitro* cytotoxicity (in HaCaT, and NIH-3T3 cells) at a concentration of 512 μg/mL. We also performed *in silico* analysis of toxicological properties and showed that EPIIS had low predicted toxicity. To confirm this, we investigated systemic acute toxicity *in vivo* by orally administering a 2000 mg/kg dose to Swiss mice. Treated mice showed no significant changes in hematological, biochemical, or histological parameters compared to non-treated animals. Epiisopilosine showed potential as a schistosomicidal drug: it did not cause acute toxicity and it displayed an acceptable safety profile in the animal model.

## Introduction

Brazilian biodiversity has been studied extensively and increasing numbers of studies related to plant species and natural resources have contributed to new therapeutic alternatives. An example of this development concerns the *Pilocarpus microphyllus* Stapf ex Wardlew species, popularly known as jaborandi, originating from North and Northeast Brazil [1,2]. Pilocarpine alkaloid is produced commercially by extraction from jaborandi leaves; the alkaloid is used commercially for eye procedures and treatment of glaucoma, and is therefore of great economic interest [3,2].

Jaborandi leaves, like other plant species, contain several bioactive metabolites whose pharmacological and physiological properties have not been fully elucidated or are still being studied; they also contain the epiisopiloturine (EPI), an alkaloid with antischistosomal activity [4, 5, 6, 7, 8].

**Fig 1:**
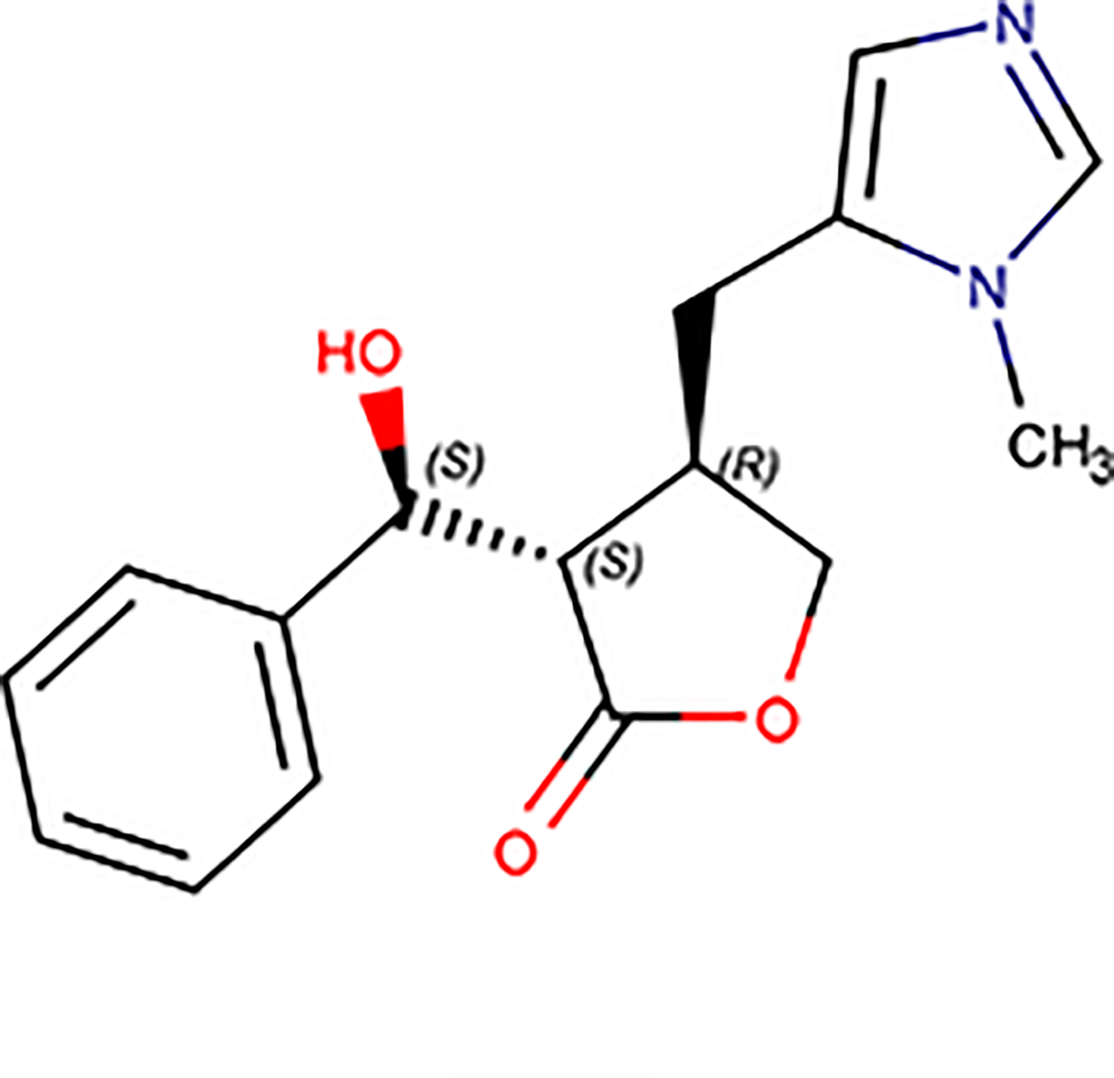
Chemical structure of EPIIS alkaloid with its heteroatoms and stereoisomer annotated. EPIIS possesses 39 atoms (C_16_H_18_O_3_N_2_).

EPIIS, or (3R,4S)3[(S)hydroxy(phenyl)methyl]4[(3methyl3H1λ^2^ imidazolidin4yl) methyl] oxolan 2 one (Fig 1), is another alkaloid from jaborandi that has been reported to act as a peripheral parasympathetic nervous system stimulant [5]. Despite few studies having focused on EPIIS biological activities, its *in vitro* activity against *S*. *mansoni* has been previously reported [2]. These results highlighted the need to perform *in vivo* studies of EPIIS, primarily regarding its possible use in the treatment of schistosomiasis. Moreover, it is important to evaluate possible toxicity, as acute toxicity studies in animals are used to fulfill various requirements related to the control of risks to human health and the environment [9,10].

In this study, we evaluated the *in vivo* antischistosomal activity of EPIIS against adult and juvenile *S*. *mansoni* worms. The effects of this alkaloid on tegument morphology were investigated via scanning electron microscopy (SEM), and effects on egg laying and hepatosplenomegaly reduction were also recorded. Furthermore, we evaluated *in vitro* cytotoxicity in different cell lines and *in silico* and *in vivo* acute toxicity.

## Materials and methods

### EPIIS purification

EPIIS alkaloid was purified from biomass generated by production of pilocarpine salts. The biomass assessment process was as previously described [11]. Alkaloids were extracted by a process based on acidification and filtration, followed alkalization [11]. EPIIS isolation was carried out using high performance liquid chromatography (HPLC) according to previously described methods [2]. Mass spectrometry was used to evaluate the purity and monoisotopic molecular mass of EPIIS (AmaZon SL, Bruker Daltonics, Bremen, Germany), acquired in a mass range of m/z 100 to 400 Da, and the positive electrospray mode was used. MS/MS was performed in manual mode with fragmentation of the precursor ion by collision induced dissociation (CID) using He as the collision gas. Precursor ions were selected within an isolation width of 2 Da and scans were accumulated with variable RF signal amplitudes [11].

### In vivo assay against *S*. *mansoni*

#### *Schistosoma* strain and hosts

We used *S*. *mansoni* BH strain Belo Horizonte, Minas Gerais, Brazil, maintained in the planorbid mollusc *Biomphalaria glabrata* as the intermediate host. For definitive hosts, *Mus musculus* female BALB/c SPF, weighing 20 g at 4 weeks of age, were previously infected using exposure to a suspension containing 70 cercariae by the tail immersion technique, as described [12]. After infection, the animals (8 per group) were maintained in vented rack system, Ventilife Alesco mini-isolators with 32 cm length per floor area (451 cm^2^), temperature 24°C, autoclaved shavings, ration feed and *ad libitum* water. For management and monitoring of animals, two weekly shavings were performed and behavior was observed until the end of the experiment. All studies were performed at the Biology Institute—Helminthology Laboratory of Neglected Diseases at the Department of Animal Biology, IB, Unicamp, São Paulo, Brazil.

#### Animal groups and *in vivo* treatments

We analyzed the effect of EPIIS in mice with adult *S*. *mansoni* infections (patent infections). In the first step, animals were divided into two groups (n = 8) and were treated 60 days post-infection (dpi). Group I animals were treated with a single dose of 400 mg/kg EPIIS, and group II animals (control group) were given 0.3 mL phosphate buffered saline (PBS). In the second step, animals were divided into two groups (n = 8) and were also treated 60 days dpi. Group III animals were treated with a single dose of 100 mg/kg EPIIS, and group IV animals (control group) were given 0.3 mL PBS. In all groups, EPIIS solubilized with PBS was administered by the oral route.

Subsequently, on the basis of their *in vivo* activity against adult schistosomes, EPIIS was tested in mice harboring juvenile *S*. *mansoni* (pre-patent infection). In this case, 21 days post infection, a group of 8 mice were treated with a single 100 mg/kg oral dose of EPIIS, and the other group of 8 untreated mice served as controls.

#### Worm recovery and treatment analysis

The analyses were performed two weeks after treatment. Mice were euthanized via cervical dislocation and adult worms were retrieved through perfusion of the hepatic portal system and mesenteric veins as described [13]. The percentage of worm reduction (WR) was calculated as described [14]. The counting of the eggs eliminated through the feces (OPG) was performed using the Kato-Katz quantitative method [15]. The percentages of the various egg developmental stages: immature, mature, and dead eggs (the oogram method) were calculated from the small intestinal wall of infected mice as described [16]. No animals died before the final trial period (75 days).

#### Scanning electron microscopy (SEM)

For microscopic analysis, a new group of animals was treated separately following the treatment scheme reported above, but with the difference that mice were euthanized via cervical dislocation 48 h after treatment. Adult *S*. *mansoni* worms were retrieved as described above. For preparation and analysis, the worms were washed with sodium cacodylate buffer (0.1 M), fixed in 2.5% glutaraldehyde (pH 7.4, Merck) for 24 h, and then fixed in 1% osmium tetroxide for 1 h. Specimens were dehydrated with increasing concentrations of ethanol (70%, 80%, 90%, and 100%) for 30 min each, dried in a critical point dryer, mounted on stubs, metalized with gold particles using a sputter coater, and finally analyzed and photographed using an electron microscope (Jeol-JSM-820).

### *In silico* analysis of toxicological properties

To analyze the toxicological parameters, we first needed to calculate LogP (lipophilicity), total polar surface area, number of hydrogen bond donors and acceptors, molecular weight and number of rotatable bonds for comparison with molecules that have similar characteristics. All these data and *in silico* toxicological properties were calculated using pkCSM software, an *in silico* tool web server maintained by VLS3D (Cambridge University) that graph-based signatures to develop predictive models of central toxicological properties for drug development [17].

### Cell culture and *in vitro* cytotoxicity assays

Murine fibroblasts (NIH-3T3) and human keratinocytes (HaCaT) cells were grown in Dulbecco’s modified Eagle’s medium supplemented with 10% fetal bovine serum and 1% antibiotic solution. The cells were maintained in an incubator under humidified atmosphere with 5% CO_2_ at 37°C. For real-time cell analysis (RTCA, xCelligence, Roche, Switzerland), the NIH-3T3 and HaCaT cells were seeded in culture plates containing electronic biosensors. After 24 h of culture, cells were treated with EPIIS or no drug (control). The concentrations of EPIIS were 32 and 512 μg/mL (equivalent to 111.8 and 1790.2 μM, respectively), and the adhered cell rates were measured every 30 min for 137 h.

### Acute toxicity evaluation

The *in vivo* toxicological evaluation of EPIIS was performed using the acute toxic class test [18], guide number 423 [9]. Female Swiss mice were divided into two groups of three animals each: a control group treated orally with a solution of 5% DMSO in saline and a group treated orally with EPIIS. The drug was solubilized at the highest recommended dose (2000 mg/kg body weight) in a solution of 5% DMSO in saline. Clinical and behavioral parameters were evaluated according to the OECD’s Guide for Recognition, Evaluation and Use of Clinical Signals [19].

#### Evaluation of hematological and biochemical parameters

After the 14th day of observation, the animals were euthanized by asphyxiation in a CO_2_ chamber and blood samples were collected from the animals via the orbital plexus. For biochemical analyses, the material was centrifuged at 1200 x *g* for 5 min before determining glucose, urea, creatinine, aspartate aminotransferase (AST), alanine aminotransferase (ALT), total cholesterol, HDL, LDL, alkaline phosphatase, total protein, albumin, and globulin levels. The values for red blood cells, leukocytes, platelets, hemoglobin, mean corpuscular volume, and mean corpuscular hemoglobin were determined immediately after collection by means of the automatic hematology Advia 120/hematology (Siemens) analyzer. Differential leukocyte counts were performed on smears stained with May-Grünwald-Giemsa [20,21].

#### Histopathological analysis

Immediately after euthanasia, fragments of liver, spleen, kidney, brain, lung, stomach, small intestine, and large intestine were collected and fixed in 10% buffered formaldehyde. After fixation, the organs were dehydrated in serial dilutions of ethyl alcohol for 1 h in each dilution, diaphanized in xylene for 30 min, and soaked in paraffin for 5 min. Sections of these organs (4 μm) were stained with hematoxylin and eosin and examined under a light microscope [22].

### Ethical conduct of research

The animals used in the *in vivo* studies were provided by Multidisciplinary Center for Biological Research (CEMIB) of the State University of Campinas. All experiments were approved by the Ethics Commission for the Use of Animals (CEUA/UNICAMP, protocol n° 2170–1), according to Brazilian Society of Science in Laboratory Animals (SBCAL) and Ethical Principles for the Use of Animals.

### Statistical analysis

GraphPad Prism (version 5.0) software was used for analyses of data significance. For therapeutic effect assays (*in vivo* assays) ANOVA and Dunnett’s test were used to analyze the statistical significance of differences between mean experimental and control values. For the RTCA assay we analyzed mean and standard deviation. A *P* value of < 0.05 was considered significant.

## Results

### EPIIS purification

**Fig 2:**
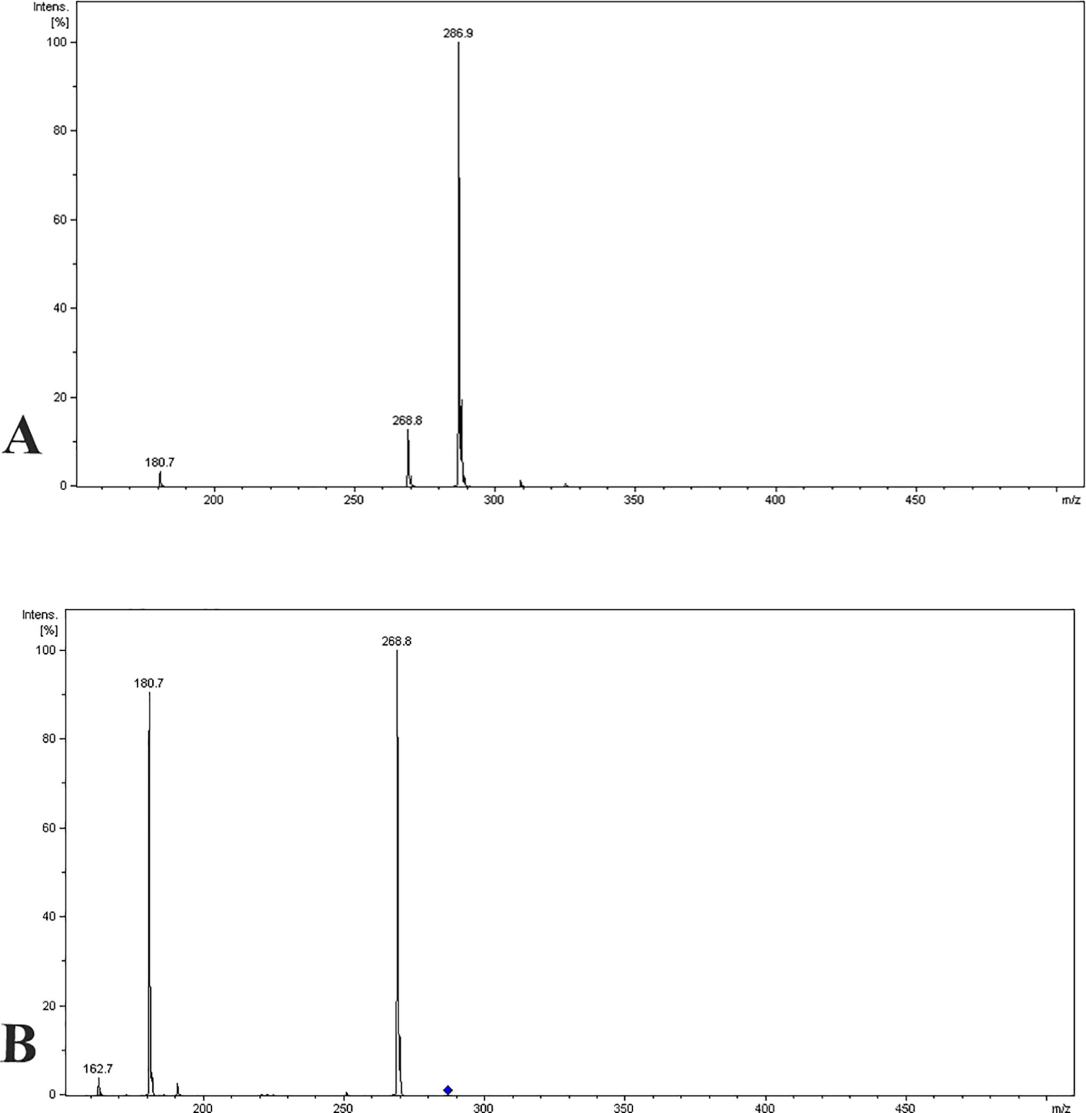
EPIIS mass spectrum. (A) pseudomolecular ion with m/z 286.9 Da [M + H]^+^. (B) MS^2^ fragments with m/z 268.8 Da [M—H_2_O + H]^+^ e 180,7 Da.

The mass spectrometry analysis showed that the separation methods used were successful. MS/MS analysis showed a pseudomolecular ion with *m/z* 286.9 Da [M + H]^+^ and a MS^2^ fragments with *m/z* 268.8 Da [M–H_2_O + H]^+^ and 180.7 Da. This ‘‘molecular fingerprint” confirmed EPIIS (Fig 2).

### EPIIS showed activity against adult *S*. *mansoni-*infected mice (patent infections)

**Fig 3:**
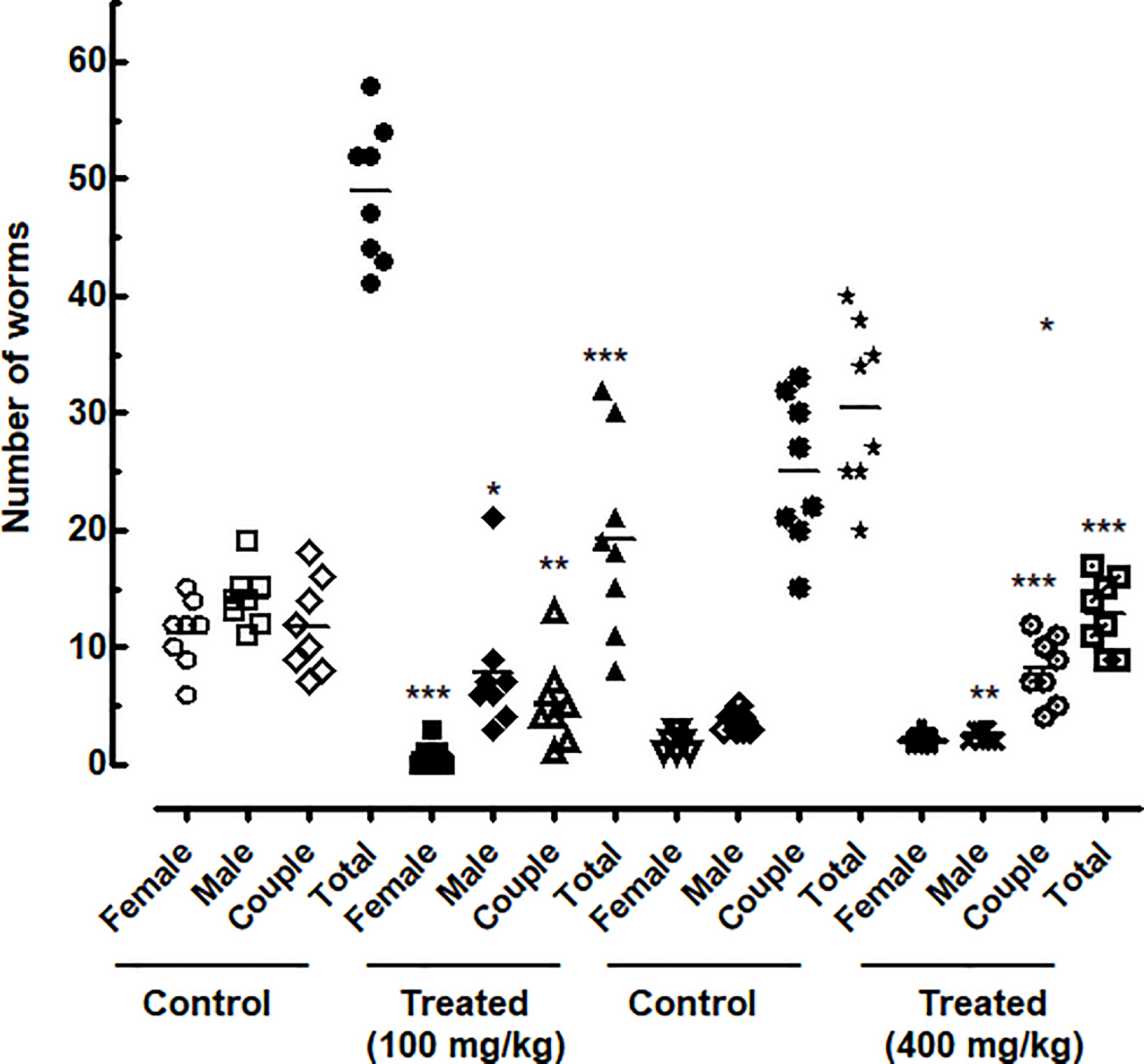
Effect on worm burden of a single oral dose of EPIIS administered to mice harboring a 60-day-old adult *S*. *mansoni* infection, stratified by sex. Points represent data from individual mice that were infected and treated with EPIIS, or infected and untreated (control). Each treated group was compared to its corresponding untreated group. The horizontal bars represent median values. *P < 0.05, **P < 0.01, ***P < 0.001 compared with untreated groups.

In mice infected by adult *S*. *mansoni*, there was a significant reduction in worm burden with oral treatment by EPIIS (Fig 3). A single dose of EPIIS of 400 mg/kg reduced worm burden by 57.78% (P < 0.001). At a dose of 100 mg/kg, the total worm burden reduction was 60.61% (P < 0.001) that of the infected untreated controls. The total, male and female worm burdens following treatment of *S*. *mansoni* infections with EPIIS are shown in Fig 3.

Because the single dose of 400 mg/kg showed no difference in terms of worm burden compared with 100 mg/kg dose, we showed the results for eggs production, liver and spleen weights, and scanning electron microscopy investigations only for the lowest dose investigated (100 mg/kg).

### EPIIS disrupted egg development as well as liver and spleen weights in mice harboring patent infections

**Fig 4:**
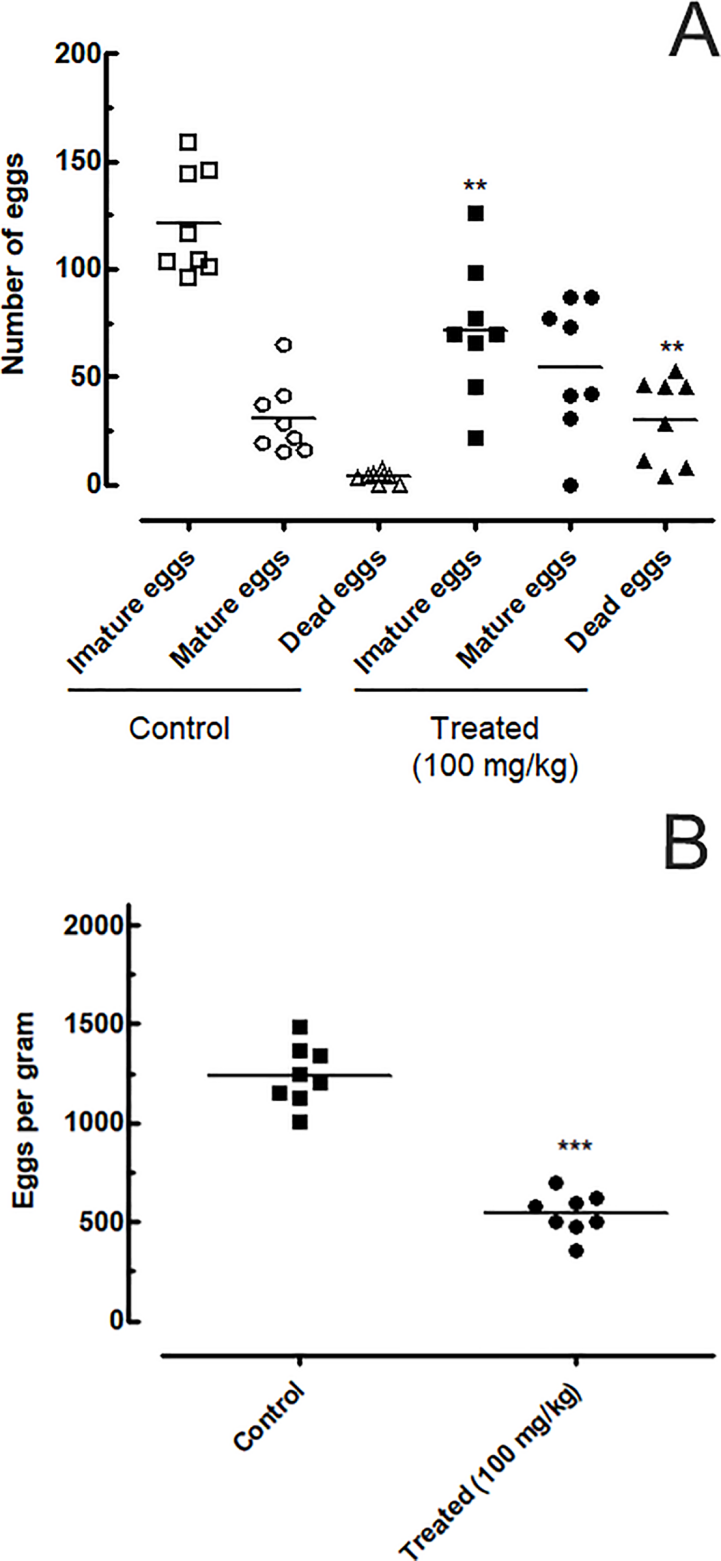
Effect on egg development and on Stoll egg count of a single dose 100 mg/kg of EPIIS administered to mice harboring a 60-day-old adult *S*. *mansoni* infection. **(A)** EPIIS effect on egg development stages (oogram) **(B)** Effect on Stoll egg count. Points represent data from individual mice that were infected and treated with EPIIS, or infected and untreated (control). The horizontal bars represent median values. **P < 0.01 and ***P < 0.001 compared with untreated groups.

The effects of EPIIS on egg development stages (oogram pattern) at 15 days after treatment are shown in Fig 4A. Administration of single dose of 100 mg/kg reduced the number of immature eggs by 45.84%. The oogram method revealed a significant increase in the percentage of dead eggs in treated mice (19.16%) compared with the control group (2.25%).

**Fig 5:**
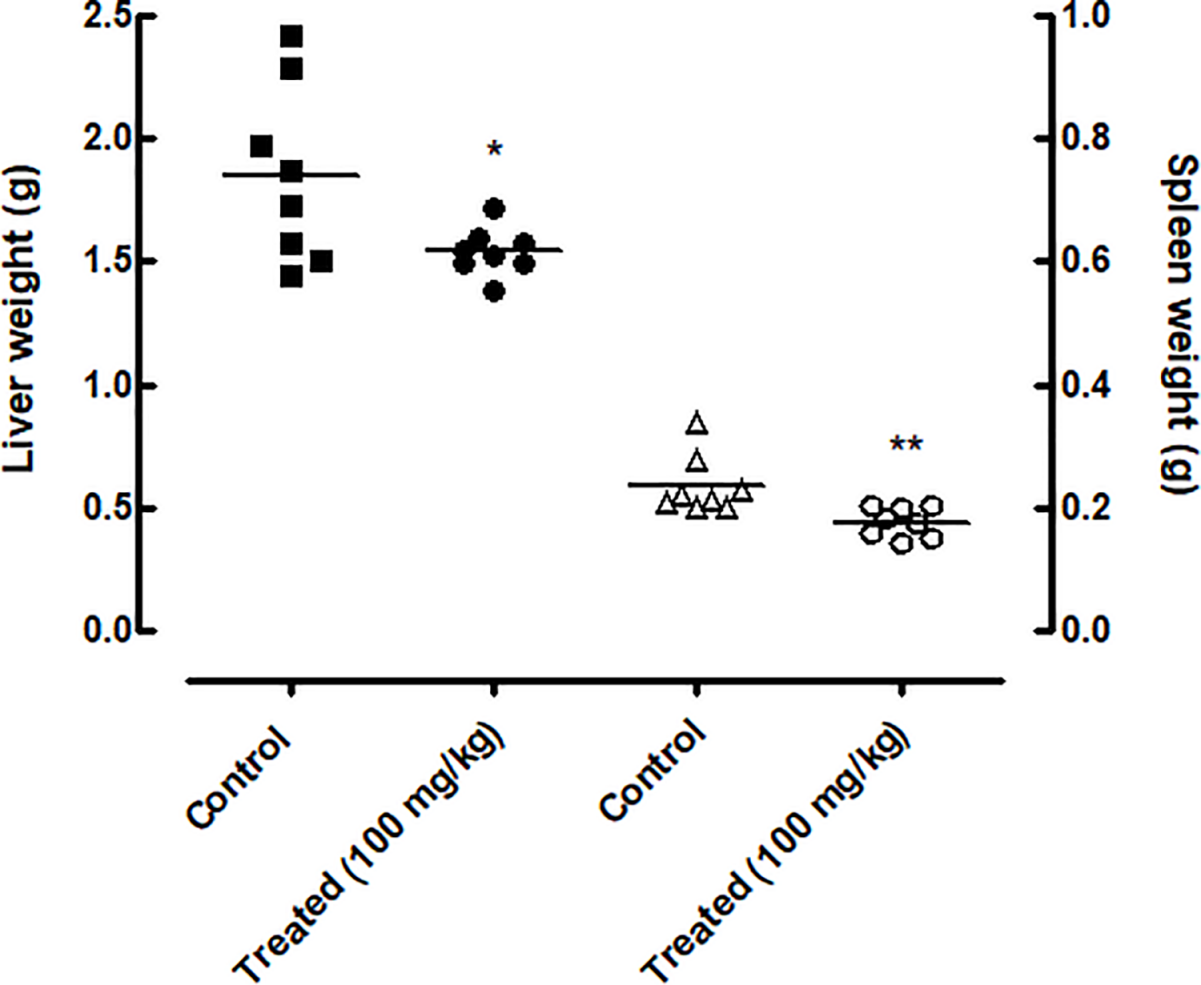
Effect of EPIIS on relative liver and spleen weights. Points represent data from individual mice that were infected and treated with EPIIS or were infected and untreated (control). The horizontal bars represent median values. *P < 0.05 and **P < 0.01 compared with untreated groups.

In feces samples collected from mice, the Kato-Katz method revealed that EPIIS significantly reduced the number of eggs compared with the control group (untreated), with a reduction of 58% (*P*<0.001) at dose of 100 mg/kg (Fig 4B). Furthermore, oral treatment with EPIIS significantly decreased hepatosplenomegaly as measured by liver and spleen weights, *P* < 0.01 and *P* < 0.05, respectively (Fig 5) compared to the control group.

### EPIIS caused changes in the tegument morphology of adult *S*. *mansoni*

**Fig 6:**
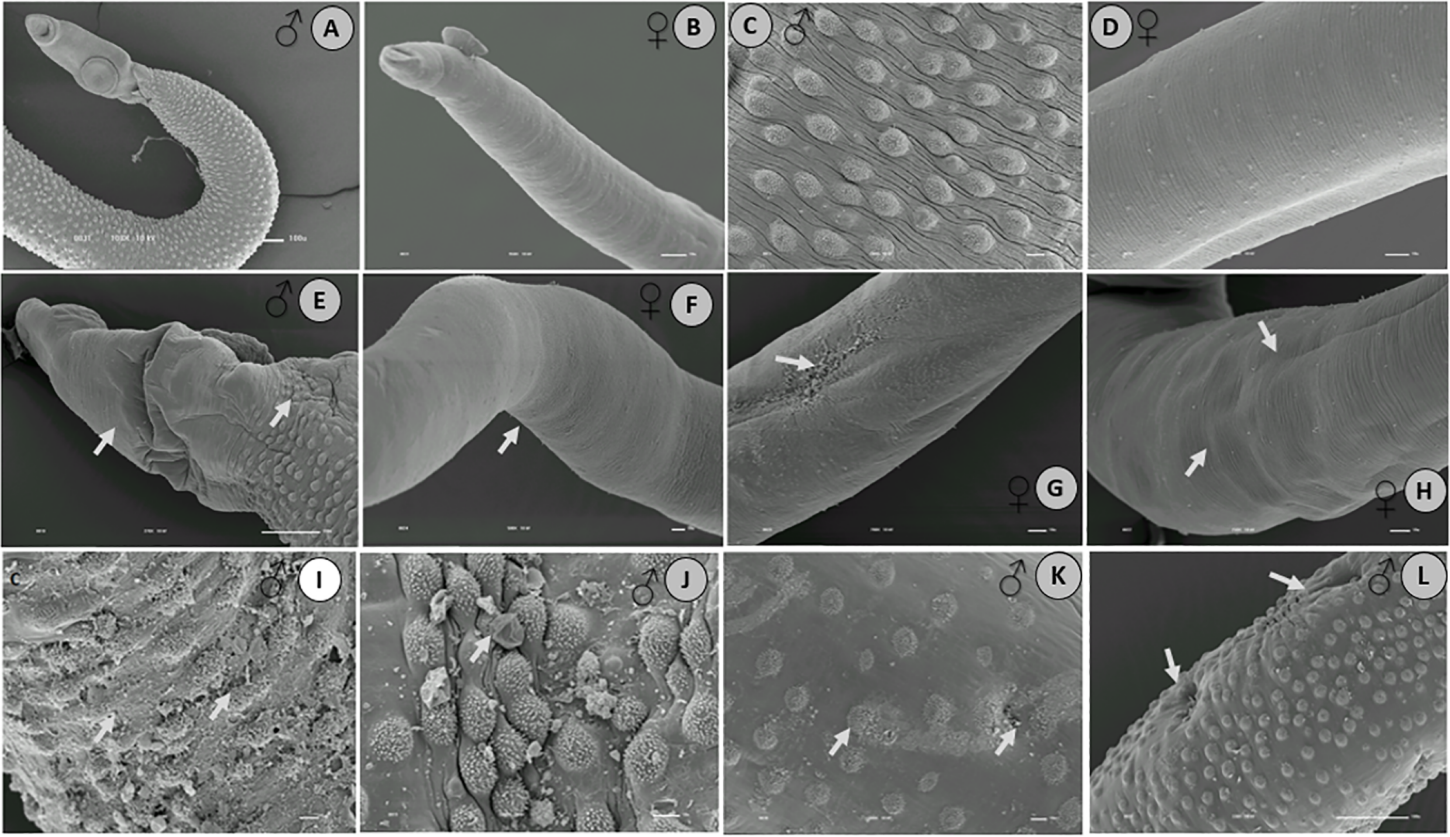
Scanning electron microscopy of *S*. *mansoni* adults retrieved 48 h after treatment with EPIIS (100 mg/kg). **A-D**: Control group—untreated; **E–L:** Male and female worms. The arrows show damage to tegument with reduction in the size of the spines, and damage to the oral and ventral suckers in male worms, followed by body corrugation in female worms. Image bar scale: (**A**) 100X___10 kv; (**B**) 120X ___10 kv; (**C-D**) 180X ___10 kv; (**E-F**) 127X ___10 kv; (**G-H**) 250X___10 kv; (**I- L)** 350X___10 kv.

In the control (untreated) group, the oral and ventral sucker, tubercles, and spines of male and female worms showed normal integrity (Fig 6A–6D). EPIIS treatment caused changes to the tegument in both males and females (Fig 6E–6L), but tegumental damage was particularly evident in male worms. The male worms showed extensive peeling of tubercles, absence of spines, and the formation of protuberances in the dorsal region (Fig 6I–6L).

### EPIIS shows moderate activity against juvenile *S*. *mansoni-*infected mice (pre-patent infections)

**Fig 7:**
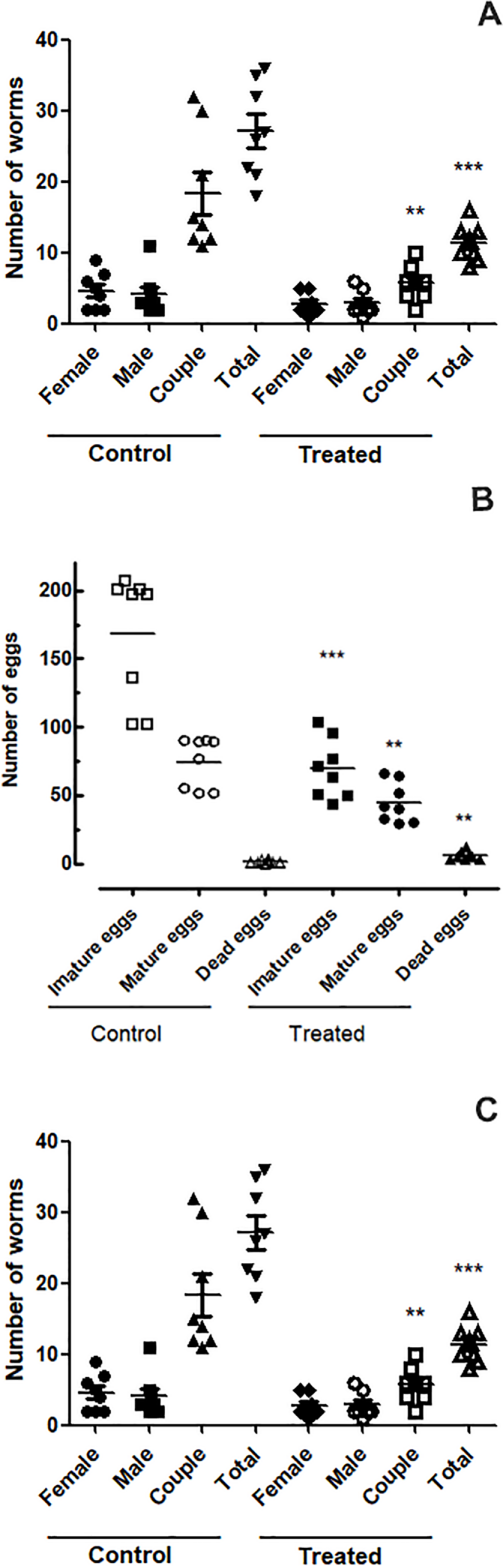
Antischistosomal effect of a single 100 mg/kg oral dose of EPIIS administered to mice harboring juvenile *S*. *mansoni* infection. **(A)** EPIIS effect on worm burden. **(B)** EPIIS effect on egg development stages (oogram) **(C)** Effect on Stoll egg count. Points represent data from individual mice that were infected and treated with EPIIS, or infected and untreated (control). The horizontal bars represent median values. **P < 0.01 and ***P < 0.001 compared with untreated groups.

Because EPIIS at dose of 100 mg/kg was the most potent against *S*. *mansoni* adults, we also analyzed the effects of EPIIS on juvenile *S*. *mansoni* worms. The treatment of juvenile *S*. *mansoni*-infected mice with single 100 mg/kg oral dose of EPIIS showed a moderate reduction in worm burden of 58.06% compared to the control group (Fig 7A). Analysis of the oogram pattern showed reductions in the number of immature eggs of 58.60% (Fig 7B). In feces samples, the Kato-Katz technique revealed egg reduction of 64.18% compared to the control group (Fig 7C).

### EPIIS gave low probability toxicity predictions

*In silico* analysis of LogP, total polar surface area, number of hydrogen bond donors and acceptors, molecular weight and number of rotatable bonds are shown in Table 1. We analyzed the predicted toxicity on human ether-à-go-go-related gene (hERG) inhibitor I and II, to evaluate whether EPIIS caused effects on cardiac repolarization. In addition, we analyzed whether EPIIS caused hepatotoxicity, because the liver is substantially affected in schistosomiasis; in addition, liver is primarily responsible for the first-pass metabolism. The human maximum tolerated dose and skin sensitization were also analyzed, because it is important to know whether the dose causes allergic responses. All results for toxicity properties are shown in Table 2.

**Table 1:** Physicochemical parameters through *in silico* analysis of EPIIS using pkCSM methodology.

Descriptor	Preferred	EPIIS
Molecular Weight (g/mol)	< 500	286.331
LogP	1–3	1.4854
Rotatable Bonds	≤ 10	4
Acceptors	≤ 10	5
Donors	≤ 5	1
Surface Area	< 140 Å^2^ (oral)	122.745

**Table 2:** *In silico* analysis of toxicological properties of EPIIS predicted using the pkCSM methodology.

Model name	Unit	Preferred	EPIIS
Max. tolerated dose (human)	log mg/kg/day	< 0.477	0.451
hERG I inhibitor	-	No	No
hERG II inhibitor	-	No	Yes
Hepatotoxicity	-	No	Yes
Skin sensitization	-	No	No

### Effect of EPIIS on cell viability

**Fig 8:**
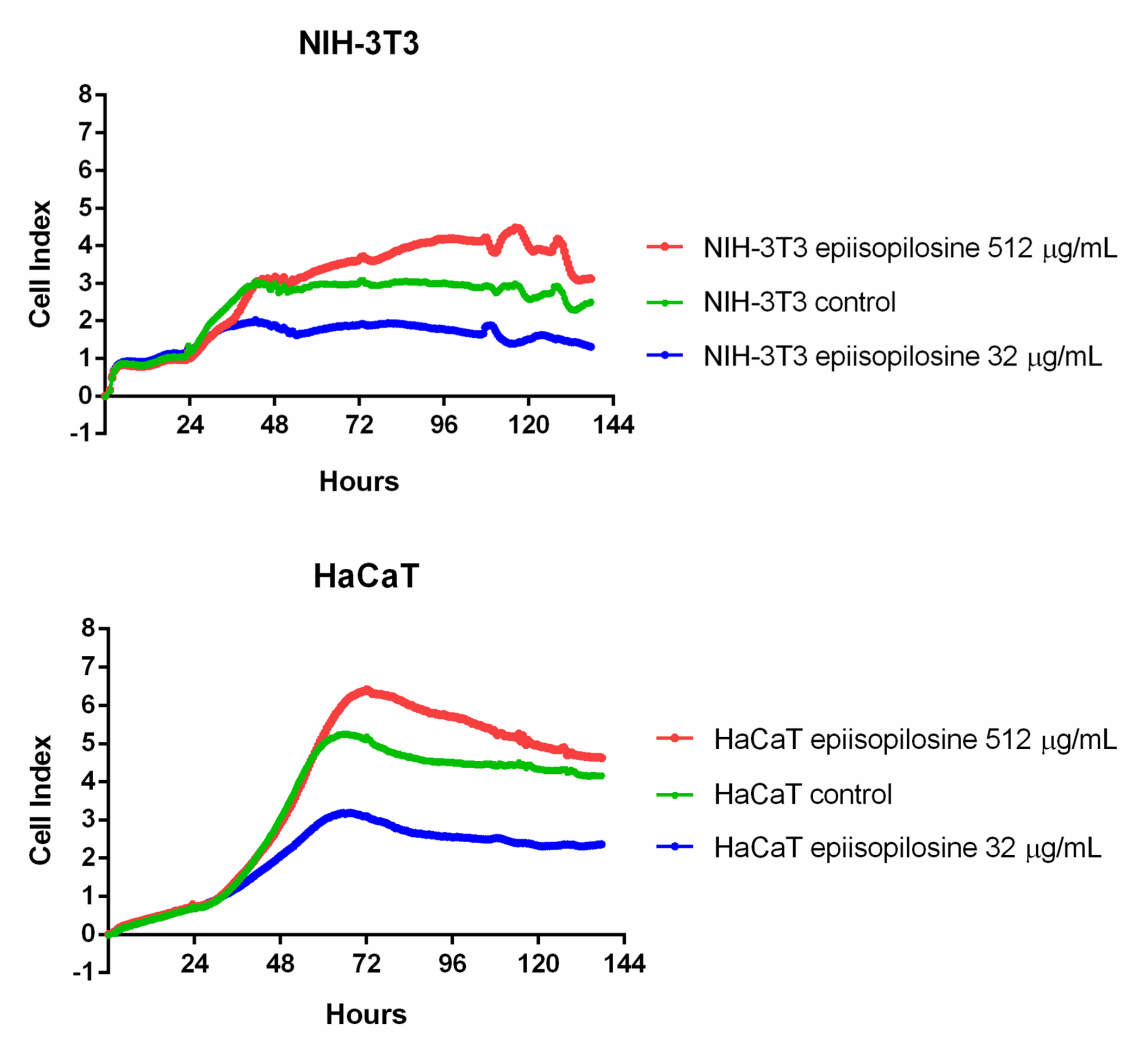
*In vitro* cytotoxicity assay with EPIIS. Adhesion indices of NIH-3T3 and HaCaT cells treated with EPIIS. The concentrations of EPIIS were 32 or 512 μg/mL and the adhered cell rates were measured every 30 min for 137 h.

Cytotoxic activity of EPIIS was determined via real-time cell index analysis (RTCA) on two normal cell lines: NIH-3T3 and HaCaT. RTCA showed that adhesion indices in both NIH-3T3 and HaCaT cells were decreased by EPIIS at a concentration of 32 μg/mL. However, no significant decrease in cell adhesion indices were found in comparison to the control groups when EPIIS was used at 512 μg/mL (Fig 8).

### EPIIS did not cause acute toxicity in mice

In acute toxicity tests in mice, no animal showed signs of toxicity (cyanosis, piloerection, contortion, eyelid ptosis, tremors, seizures, ataxia, or diarrhea) and all mice survived the 14 days examination period. Therefore, the LD_50_ was over 2000 mg/kg. Furthermore, there were no significant differences between the treated and control groups in terms of parameters of motor activity, respiratory rate, and corneal and atrial reflexes.

#### EPIIS did not cause biochemical or hematological changes in mice

The analysis of hematological parameters is more accurate for predicting the toxic effects of drugs in humans when the data are translated from animal studies [23, 24]. The hematological parameters of mice treated with EPIIS were all within the reference range (Table 3). The biochemical parameters are shown in Table 4. Total cholesterol and high-density lipoprotein (HDL) were significantly higher in the EPIIS treatment group than in the control group (*P* < 0.05 and *P* < 0.001, respectively).

**Table 3:** Hematological parameters of Swiss mice treated with EPIIS by oral route.

Parameters	Control	EPIIS
RBC (x 10/μL)	9.34 ± 0.0284	9.84 ± 0.287
Hemoglobin (g/dL)	13.9 ± 0.148	14.6 ± 0.410
Globular volume (%)	43.0 ± 0.365	44.8 ± 1.49
MCV (fL)	45.8 ± 0.121	45.6 ± 0.711
MCHC (%)	32.3 ± 0.347	32.5 ± 0.634
Total leukocyte count (/μL)	10.0 ± 0.479	10.8 ± 0.856
Lymphocytes (/μL)	8.90 ± 0.443	7.90 ± 1.60
Eosinophils (/μL)	0.124 ± 0.0394	0.0243 ± 0.0243
Monocytes (/μL)	0.100 ± 0.00479	0.101 ± 0.0538
Platelets (thousand/μL)	1030 ± 50.9	944 ± 72.6

Values are presented as means ± SEM. Number of animals/group = 6. RBC: Red blood cells; MCV: mean corpuscular volume; MCHC: mean corpuscular hemoglobin concentration.

**Table 4:** Biochemical parameters in sera obtained from Swiss mice treated with EPIIS by oral route.

Parameters	Control	EPIIS
ALT (UI/L)	83.3 ± 10.5	122 ± 25.6
AST (UI/L)	111 ± 13.6	96.2 ± 11.6
Glucose (mg/dL)	164 ± 2.14	150 ± 7.07
Urea (mg/dL)	83.3 ± 7.25	89.2 ± 6.49
Creatinine (mg/dL)	0.167 ± 0.0422	0.317 ± 0.0749
Total Cholesterol (mg/dL)	91.7 ± 2.32	138 ± 16.9*
HDL (mg/dL)	27.3 ± 2.95	55.2 ± 4.71***
LDL (mg/dL)	72.7 ± 8.33	61.7 ± 21.2
Alkaline Phosphatase (UI/L)	85.0 ± 15.5	103 ± 8.17
Total Protein (g/dL)	5.23 ± 0.0558	6.55 ± 0.742
Albumin (g/dL)	1.96 ± 0.0623	2.16 ± 0.141
Globulin (g/dL)	3.27 ± 0.0269	4.39 ± 0.621

Values are presented as means ± SEM. Number of animals/group = 6.

Asterisks indicate that differences compared to the control were significant with ***P < 0.001, and *P < 0.05. ALT: alanine transaminase; AST: aspartate transaminase; HDL: high-density lipoprotein: LDL: low-density lipoprotein.

#### EPIIS did not cause histopathological changes

**Fig 9:**
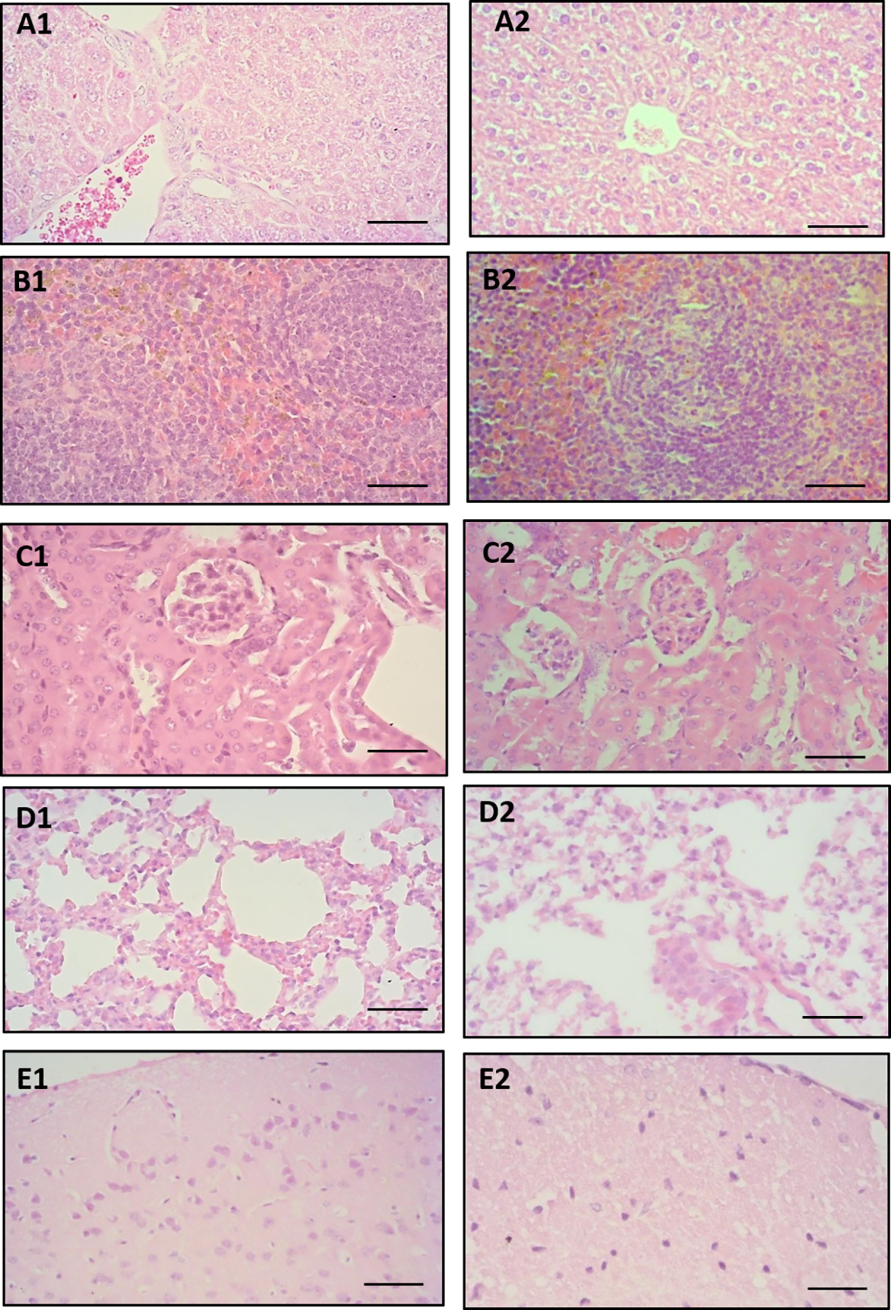
**Photomicrographs of histological sections of liver (A1, A2), spleen (B1, B2), kidney (C1, C2), lung (D1, D2) and brain (E1, E2) obtained from Swiss mice of control group (first column) and group treated with 2000 mg/kg EPIIS (second column).** Hematoxylin & eosin, 400 X magnification, bars = 50 μm.

A detailed study of histological sections of liver, spleen, brain, lung, stomach, small intestine, and large intestine from Swiss mice treated with oral doses of 2000 mg/kg of EPIIS revealed no morphological changes compared to the control group. No pathogenic changes characteristic of toxicity were found. Representative photomicrographs of histological sections of the liver, spleen, and kidney are shown in Fig 9.

## Discussion

Our data suggested that the methods used for EPIIS purification were successful because the fragments found on mass spectrometry were of a molecular structure that accorded with previously reported structures [2].

Our mouse model of schistosomiasis showed that EPIIS affected adult *S*. *mansoni* survival, inducing tegumental alterations in the oral and ventral suckers of parasites. EPIIS also affected egg production. Unlike praziquantel, which acts primarily against the adult worm, oral treatment with EPIIS also showed moderate efficacy against juvenile *S*. *mansoni*.

The worm’s tegument is well-recognized as an important drug target and study model in schistosomiasis [25,26]. Apart from having a direct effect on survival, tegumental alterations might result in exposure of parasite antigens to the host immune system [27]. According to Xiao et al. [28], damage to the suckers may result in loss of ability to adhere to blood vessels, rendering ingestion of nutrients from the blood more difficult. Other authors observed similar tegumental alterations after evaluating various compounds *in vivo*, e.g., EPI and phytol [8, 29, 30]. EPIIS has effects similar to those of pilocarpine on peripheral parasympathetic nervous system [5]. Thus, as pilocarpine was previously reported to exert no cholinergic effect on the neuromuscular system of schistosomes [31], it could be that EPIIS also does not exert this action on *Schistosoma*. The mechanism of parasite killing by EPIIS may be associated with high titers of serum antibodies against tegumental antigens; serum antibodies from mice can bind the surface membrane of EPIIS-exposed adult worms, as was shown for arachidonic acid [32].

Our results also showed a significant decrease in the total number of eggs, both in terms of the percentage of immature eggs and the increase in the percentage of mature and dead eggs. Thus, EPIIS could affect the fecundity of the worms, especially female worms, decreasing their ability to lay eggs. We may suspect direct ovicidal action, taking into account the increase in the number of dead eggs [29,33]. The results presented here regarding egg reduction were also observed by Guimarães et al. (2015) [8] in a study on the effects of the alkaloid EPI on *S*. *mansoni* at a concentration of 100 mg/kg. Our results revealed that treatment with 100 mg/kg EPIIS caused a reduction in total worm burden. In the literature however, we note that the majority of compounds tested against *S*. *mansoni* showed activity at doses higher than 100 mg/kg, e.g., dihydroartemisinin [34], and nerolidol [30], whereas (-)-6,6'-dinitrohinokinin reduced total worm burden at a concentration of 100 mg/kg [35] but to a lesser extent than did EPIIS in the present study. In addition, the treatment produced significant reduction in female worms; this is important as the females are responsible for oviposition, producing about 300 eggs/day, all of which are viable, metabolically active, and highly antigenic [36]. Our observation of higher activity of EPIIS on female *S*. *mansoni* worms accords with results from a previous *in vivo* study investigating natural antischistosomal products and natural product-derived compounds such as dihydroartemisinin [34]. We therefore speculate that EPIIS may act on different targets in males and females, however, the mechanism of action remains to be elucidated.

In addition to the therapeutic evaluation of EPIIS against *S*. *mansoni*, we performed *in vitro* cytotoxicity tests. These assays are essential in the early stages of drug development as they provide an estimation of the starting dose and range of concentrations to be used in *in vivo* non-clinical stages [10, 37]. Our results accord with findings reported for other imidazole compounds that did not demonstrate strong toxic activity in mammalian cells (acute toxicity and cytotoxicity) [38]. Interestingly, among all the EPIIS concentrations tested on non-tumoral fibroblast cells, 32 μg/mL was the only concentration that induced reduction in cell viability. It is known that poorly water-soluble molecules tend to form aggregates and/or precipitates depending on various factors including physicochemical properties, concentration, pH, salt, and ionic strength [39,40]. This phenomenon may be exemplified by phthalocyanines (hydrophobic photosensitizers used for medical applications) where the increase of their concentration in aqueous media led to aggregate formation that impaired their effectiveness [41]. We suggest that EPIIS, also a hydrophobic molecule, had a similar dose-dependent behavior in the *in vitro* assays because significant cytotoxicity was observed with a 32 μg/mL EPIIS treatment while no cytotoxicity was shown with the dose of 512 μg/mL (16x higher). Detailed analysis of EPIIS behavior in solution should be considered in further studies.

However, some azoles, such as ketoconazole, display high renal and hepatic toxicity by binding to mammalian P450 enzymes, including P450 3A4 and several steroid hydroxylases [42]. Thus, because of the known toxicity of some alkaloids, we performed a toxicity prediction study and an acute toxicity study of mice treated with EPIIS.

The *in silico* analysis revealed that physicochemical parameters of EPIIS were within preferred specifications. The predicted toxicity showed that EPIIS had a human maximum tolerated dose with value within preferred specifications. The pkCSM prediction of hERG inhibition considers the IC_50_ values of about 360 compounds (for hERG I analysis) and molecular parameters of more 800 compounds (for hERG II analysis) [10, 43,44]. In our study, the hERG II model, but not the hERG I model showed positive results. These data point to a possible cardiotoxic effect of EPIIS, but as different datasets were applied in the same model, the prediction results may show some variability, as was shown for the BPP-brachyNH2 peptide [10]. More investigations are needed in this respect in view of the known parasympathomimetic action of the drug on vertebrate heart and vascular smooth muscles. In addition, although the *in silico* study predicted hepatotoxicity for EPIIS, no alteration was observed in the *in vivo* serum biochemical parameters, including aspartate aminotransferase (AST), alanine aminotransferase (ALT), as well as in the liver histopathological analysis.

With respect to acute toxicity, EPIIS was considered safe, with LD_50_ above 2000 mg/kg. In addition, all hematological parameters for mice in EPIIS treatment group were within the reference ranges, indicating that the tested alkaloid may not have harmful effects on bone marrow function and may not induce anemia. Furthermore, we observed no marked alterations in serum biochemical parameters of treated animals compared to those of control animals. However, more studies are needed to understand the effect of EPIIS on increased cholesterol and HDL values, although the increase in lipoprotein can be beneficial in hypercholesterolemia patients. In addition, the increase in total cholesterol may have occurred due only to the EPIIS effect on HDL fraction. Corroborating with the biochemical results obtained for this study, there were no significant histopathological changes or morphological differences in analyzed organs (liver, spleen, kidneys, brain, lungs, heart, stomach, small intestine, and large intestine) for mice in the control group and those treated with EPIIS.

In conclusion, the present study results demonstrated that the main action of EPIIS was on female worm burden. In addition, the treatments caused significant reduction in egg burden and morphological changes in the parasites, parasitological parameters essential for drug discovery for schistosomiasis. The alkaloid did not show *in vitro* cytotoxicity against the cell lines tested at a concentration of 512 μg/mL, nor *in vivo* toxicity following single dose administration in the experimental model. Thus, the absence of toxicity observed for this alkaloid demonstrated an acceptable safety profile for studies in animal models aiming to investigate the pharmacological, biotechnological, and therapeutic potential of this molecule related to its activity against schistosomiasis.

## Supporting information

S1 Dataset NC3Rs ARRIVE guidelines checklist. (PDF)
